# Long Noncoding RNA FAM201A Mediates the Radiosensitivity of Esophageal Squamous Cell Cancer by Regulating ATM and mTOR Expression via miR-101

**DOI:** 10.3389/fgene.2018.00611

**Published:** 2018-12-05

**Authors:** Mingqiu Chen, Pingping Liu, Yuangui Chen, Zhiwei Chen, Minmin Shen, Xiaohong Liu, Xiqing Li, Anchuan Li, Yu Lin, Rongqiang Yang, Wei Ni, Xin Zhou, Lurong Zhang, Ye Tian, Jiancheng Li, Junqiang Chen

**Affiliations:** ^1^Department of Radiation Oncology, Fujian Medical University Union Hospital and Fujian Provincial Platform for Medical Laboratory Research of First Affiliated Hospital, Fujian, China; ^2^Department of Radiation Oncology, The Second Affiliated Hospital of Soochow University, Suzhou, China; ^3^Institute of Radiotherapy & Oncology, Soochow University, Suzhou, China; ^4^Shengli Clinical Medical College, Fujian Medical University, Fuzhou, China; ^5^Department of Radiation Oncology, Fujian Medical University Union Hospital, Fuzhou, China; ^6^Fuzhou Center for Disease Control and Prevention, Fuzhou, China; ^7^Department of Radiation Oncology, Fujian Cancer Hospital & Fujian Medical University Cancer Hospital, Fuzhou, China; ^8^Cancer and Genetics Research Complex, Department Molecular Genetics and Microbiology, College Medicine, University of Florida, Gainesville, FL, United States

**Keywords:** ATM, esophageal squamous cell carcinoma, FAM201A, long noncoding RNA, miR-101, mTOR, radiosensitivity

## Abstract

**Background:** The aim of the present study was to identify the potential long non-coding (lnc.)-RNA and its associated molecular mechanisms involved in the regulation of the radiosensitivity of esophageal squamous cell cancer (ESCC) in order to assess whether it could be a biomarker for the prediction of the response to radiotherapy and prognosis in patients with ESCC.

**Methods:** Microarrays and bioinformatics analysis were utilized to screen the potential lncRNAs associated with radiosensitivity in radiosensitive (*n* = 3) and radioresistant (*n* = 3) ESCC tumor tissues. Reverse transcription-quantitative polymerase chain reaction (RT-qPCR) was performed in 35 ESCC tumor tissues (20 radiosensitive and 15 radioresistant tissues, respectively) to validate the lncRNA that contributed the most to the radiosensitivity of ESCC (named the candidate lncRNA). MTT, flow cytometry, and western blot assays were conducted to assess the effect of the candidate lncRNA on radiosensitivity *in vitro* in ECA109/ECA109R ESCC cells. A mouse xenograft model was established to confirm the function of the candidate lncRNA in the radiosensitivity of ESCC *in vivo*. The putative downstream target genes regulated by the candidate lncRNA were predicted using Starbase 2.0 software and the TargetScan database. The interactions between the candidate lncRNA and the putative downstream target genes were examined by Luciferase reporter assay, and were confirmed by PCR.

**Results:** A total of 113 aberrantly expressed lncRNAs were identified by microarray analysis, of which family with sequence similarity 201-member A (FAM201A) was identified as the lncRNA that contributed the most to the radiosensitivity of ESCC. FAM201A was upregulated in radioresistant ESCC tumor tissues and had a poorer short-term response to radiotherapy resulting in inferior overall survival. FAM201A knockdown enhanced the radiosensitivity of ECA109/ECA109R cells by upregulating ataxia telangiectasia mutated (ATM) and mammalian target of rapamycin (mTOR) expression via the negative regulation of miR-101 expression. The mouse xenograft model demonstrated that FAM201A knockdown improved the radiosensitivity of ESCC.

**Conclusion:** The lncRNA FAM201A, which mediated the radiosensitivity of ESCC by regulating ATM and mTOR expression via miR-101 in the present study, may be a potential biomarker for predicting radiosensitivity and patient prognosis, and may be a therapeutic target for enhancing cancer radiosensitivity in ESCC.

## Introduction

Globally, esophageal cancer (EC) is one of the most common types of cancer, with the 7th highest incidence rate and 6th greatest rate of cancer-associated death (Bray et al., 2018). Surgery still plays an important role in the treatment of EC (Pennathur et al., 2013; Rustgi and El-Serag, 2014). However, due to the patients' physiological conditions, the tumor location or the tumor stage, only ~25% of newly diagnosed patients are suitable for surgery (Short et al., 2017). For patients with unresectable EC, radiotherapy (RT) combined with chemotherapy is considered to be the optimal treatment (Sasaki and Kato, 2016).

However, predominantly because of local failure (Lloyd and Chang, 2014; Versteijne et al., 2014) which has been associated with intrinsic and/or acquired radioresistance (Chen X. et al., 2017), the survival rate in EC patients following RT is as low as 10–30% after 5 years (Cooper et al., 1999; Gwynne et al., 2011). Therefore, how to predict the radiosensitivity and resensitize patients is imperative in patients with EC treated with RT. Unfortunately, as the molecular mechanism of radioresistance, which is known to involve DNA repair proteins (Zafar et al., 2010), cell signal pathways (Dumont and Bischoff, 2012), angiogenesis (Francescone et al., 2011), cancer stem cells (Moncharmont et al., 2012), and autophagy (Chaachouay et al., 2011), is intricate and has not been elucidated thoroughly, there are currently no accurate biomarkers to predict radioresistance or therapeutic targets to enhance the radiosensitivity of EC.

Long non-coding RNAs (lncRNAs) are a new class of non-protein-coding transcripts that are longer than 200 nucleotides (Qi and Du, 2013). A number of previous studies have demonstrated that lncRNAs are important regulators of gene expression, that control both physiological and pathological processes in development and diseases such as cancer (Kung et al., 2013). Recent studies have reported that lncRNAs also function as regulators of tumor radiosensitivity and may serve as biomarkers for tumor response to RT (Spizzo et al., 2012; Yu et al., 2012). However, radiosensitivity-associated lncRNAs in esophageal squamous cell carcinoma (ESCC) are rarely reported (Tong et al., 2014; Zhang et al., 2015; Li et al., 2016; Zhou et al., 2016).

In the present study, we demonstrated that the lncRNA family with sequence similarity 201-member A (FAM201A) contributed the most to the radioresistance of ESCC. Furthermore, functional and mechanistic analyses revealed that FAM201A contributed to radioresistance by upregulating ataxia telangiectasia mutated (ATM) and mammalian target of rapamycin (mTOR) expression via actions as a miR-101 sponge. This study first established a FAM201A-miR-101-ATM/mTOR regulatory network in ESCC, revealing a promising therapeutic strategy for treating ESCC with radioresistance.

## Materials and Methods

### Patients and Tissue Specimens

The present study was approved by the Fujian Medical University Union Hospital Institutional Review Board (No. 2014KY001). All of the patients signed informed consent prior to treatment, and all of the information was anonymized prior to its analysis. The pretreatment work-up and eligibility criteria, details of radiotherapy and chemotherapy, criteria for toxicity, and short-term response, follow-up and the statistical analysis of survival were presented in our previous study (Chen M. Q. et al., 2017).

Between July 2015 and March 2017, a total of 41 patients with ESCC who received RT were recruited. Tissue specimens obtained during pretreatment with esophagogastroduodenoscopy were histopathologically examined by two independent pathologists and were snap frozen in liquid nitrogen and then stored at −80°C until RNA extraction.

Tissue specimens were divided into a radiosensitive group (*n* = 23) and a radioresistant group (*n* = 18) based on short-term response to RT. The short-term responses to RT were classified as a clinically complete response (CR), partial response (PR), stable disease (SD), or progressive disease (PD) according to the Japanese Classification of Esophageal Cancer guidelines (Japan Esophageal Society, 2017). Of these, the CR and PR were termed radiosensitive group and the SD and PD were termed radioresistant group in the current study.

### Microarray Screening and Bioinformatics Analysis

Microarray profiling was performed using three radiosensitive ESCC tumor tissues and three radioresistant ESCC tumor tissues. RNA extraction and sequential microarray hybridization were conducted by Biotechnology Company (Shanghai, China), and the detected human genome transcripts were obtained by the Human lncRNA array V6.0 (4x180 K; Agilent Technologies, Inc., Santa Clara, CA, USA). Bioinformatics analysis was performed using GeneSpring Software to obtain differentially expressed lncRNAs correlated with ESCC radiosensitivity.

### Cell Lines and Culture

The ESCC cell line Eca109 was obtained from Chinese Academy of Sciences (Beijing, China). The corresponding radioresistant cells (Eca109R) were established from the parental cell line Eca109 by stepwise X-ray irradiation at 30 Gy in three fractions (10 Gy per fraction) (Da et al., 2017). Cells were cultured in RPMI-1640 medium (HyClone; GE Healthcare Life Sciences, Logan, UT, USA) with 10% (v/v) fetal bovine serum (Thermo Fisher Scientific, Inc., Waltham, MA, USA) and antibiotics (100 U/mL penicillin and 100 μg/mL streptomycin; HyClone) in an atmosphere of 95% air/ 5% CO_2_ at 37°C.

### RNA Isolation and Reverse Transcription-Quantitative Polymerase Chain Reaction (RT-qPCR)

Total RNAs from either tissue samples or cultured cells were extracted with TRIzol reagent (Thermo Fisher Scientific, Inc.) according to the manufacturer's instructions. The RNA concentration and quality were measured using a NanoDrop ND-2000 spectrophotometer which measured the absorbance at 260 and 280 nm. Samples with an A_260_:A_280_ ratio ≥2.0 were selected for further analysis.

First strand cDNA for the potential lncRNAs and putative micro (mi)-RNA were synthesized using the PrimeScript^TM^ RT reagent kit with gDNA Eraser (Takara Biotechnology, Co., Ltd., Dalian, China) according to the manufacturer's protocol. Briefly, 1 μg total RNA, 2 μl 5X gDNA Eraser Buffer, 1 μl gDNA Eraser and RNase Free dH_2_O, were combined in a total reaction volume of 10 μl and incubated at 42°C for 2 min to eliminate the genomic DNA. A total of 10 μl of the RT reaction mixture (consisting of 4 μl 5X PrimeScript Buffer 2, 1 μl PrimeScript RT Enzyme Mix 1, 1 μl RT Primer Mix, and 4 μl RNase Free dH_2_O) was then added, and the mixture was incubated at 37°C for 15 min, followed by 85°C for 5 s to generate the cDNA.

The expression of the potential lncRNAs in the radiosensitive tumor tissues, compared with the radioresistant tumor tissues, was quantified using SYBR^Ⓡ^ Premix Ex Taq (Takara Biotechnology Co., Ltd.) according to the manufacturer's instructions on the ABI 7500 Real-Time PCR System (Applied Biosystems; Thermo Fisher Scientific, Inc.). Briefly, the 20 μl reaction mixtures were incubated at 95°C for 30 s for the initial denaturation, followed by 40 cycles at 95°C for 5 s and 60°C for 34 s. The expression levels of lncRNAs were calculated using the ΔCt method, where ΔCt = Ct _target_ -Ct _reference_, a smaller ΔCt value indicates a greater expression. The relative expression of lncRNAs was analyzed using the 2^−ΔΔ*Ct*^ method (Livak and Schmittgen, 2001); data was normalized to the endogenous control GAPDH. Each sample was examined in triplicate. The primers and oligonucleotides of the plasmid were synthesized by Invitrogen (Thermo Fisher Scientific, Inc.), the sequences are presented in Table 1. The aberrant lncRNA that had the greatest sensitivity and specificity for predicting ESCC radiosensitivity (in radiosensitive and radioresistant tissues), as identified by receiver operating characteristic (ROC) curves, and was associated with survival, was identified as the candidate lncRNA for further study.

**Table 1:** The primer sequences used in reverse transcription-quantitative polymerase chain reaction.

**Primers used for RT-qPCR**	**Forward (5^′^-3^′^)**	**Reverse (3^′^-5^′^)**
FAM201A	TCTCTGATGGGAGCCTCTTTA	CAAGCCACAGACGGAGAAA
CASC2	GTCCGCATGGTAAGGAATCA	GACTGCGTTTATCAAGTCCAAAG
DLEU2	TGGCGCAGTCGGTTTAAT	TTCCTTGCAGTACACCTTTCA
DLX6-AS1	TCTCCTCCTACCTAGCATCTTC	CCTTTGAAGCTCCTACTCCTTT
MCF2L-AS1	TTGAGCCTGGGCAATGTAG	CTTCCTGCTGGAATTCTCTCTC
GAPDH	CAGGGCTGCTTTTAACTCTGGTAA	GGGTGGAATCATATTGGAACATGT
FAM201A mimic	GGGGTACCGAGTGCACCTGGCCTGAGAG	GGAAGCCTTTTGTGGTTAGATATTTGAAAT
**OLIGONUCLEOTIDES OF PLASMID**		
siFAM201A	GATCTTTCGTCCATTTACTtt
NC-siFAM201A	GCCTTATTTCTATCTTACGtt
FAM201A-cDNA	GTACCTCGATCTTTCGTCCATTTACTTCAAGAGA GTAAATGGACGAAAGATCTTTTTGGAAA	AGCTTTTCCAAAAAGATCTTTCGTCCATTTACTCT CTTGAAGTAAATGGACGAAAGATCGAG
NC-FAM201A-cDNA	GTACCTCGCCTTATTTCTATCTTACGTCAAGAGC GTAAGATAGAAATAAGGCTTTTTGGAAA	AGCTTTTCCAAAAAGCCTTATTTCTATCTTACGCT CTTGACGTAAGATAGAAATAAGGCGAG
miR-101	AAGUCAAUAGUGUCAUGACAU
miR-590	GACGUGAAAAUACUUAUUCGAG
Negative control	UUCUCCGAACGUGUCACGUUU

### Transient Transfection

Small interfering RNA (siRNA) specifically targeting candidate lncRNA (si-candidate-lncRNA) and putative-miRNA, negative control (NC) si-candidate-lncRNA and si-putative-miRNA, candidate-lncRNA mimic, putative-miRNA mimic, and the inhibitor control were constructed by Nanjing Dongji Biotechnology Company (Nanjing, China). Ectopic expression of the candidate lncRNA was achieved by introducing the candidate lncRNA sequence into a pcDNA3.1 vector (Thermo Fisher Scientific, Inc.). Eca109/Eca109R cells were seeded into 6-well plates at a density of 1 × 10^6^ cells/well and cultured overnight prior to transfection. Then, transient transfection with oligonucleotides or plasmids into Eca109/Eca109R cells was performed using Lipofectamine 2000^TM^ (Thermo Fisher Scientific, Inc.). Cells were harvested 48 h post-transfection for subsequent analysis. PCR was used to validate the efficacy of Eca109/Eca109R cell transfection with si-candidate-lncRNA and candidate-lncRNA-mimic.

### Western Blot Analysis

Protein samples from tissues or cells were subjected to 10% SDS-PAGE and transferred to PVDF membranes. Following blocking in 5% skim milk for 2 h, the membranes were incubated overnight at 4°C with the primary antibodies against P-glycoprotein (P-gp; 1:1,000), glutathione S-transferase π (GST-π; 1:500), ATM (1:750), mTOR (1:1,000), and β-actin (1:5,000) purchased from Zen Bioscience Biotechnology, Inc. (Chengdu, China), followed by incubation with horseradish peroxidase-conjugated goat anti-rabbit secondary antibodies for 2 h (1:5,000). The antigen-antibody complexes were visualized using chemiluminescence.

### Radiosensitivity Assay

Radiosensitivity was assessed by 4-5-dimethylthiazol-2-yl)-2,5-diphenyl tetrazolium bromide (MTT) assay. ECA109/ECA109R cells (5,000/well) were incubated for 48 h prior to exposure to various doses of radiation (0 Gy, 2 Gy, 4 Gy, 6 Gy, 8 Gy, and 10 Gy). Subsequently, 10 μl of 5 mg/mL MTT was added to each well for a further 3 h, followed by the addition of 150 μl DMSO to dissolve the generated formazan crystals. The absorbance at a wavelength of 570 nm was detected using a microplate reader.

### Flow Cytometry Analysis of Apoptosis

ECA109/ECA109R cells (5,000/well) were incubated for 48 h prior to exposure to various doses of radiation (0 Gy, 2 Gy, 4 Gy, 6 Gy, 8 Gy, and 10 Gy). The ratio of apoptotic cells was detected using an Annexin V-FITC Apoptosis Detection Kit (BD Bioscience, Franklin Lakes, NJ, USA) and analyzed using a BD Calibur flow cytometer with CellQuest software (BD Biosciences).

### Candidate lncRNA Downstream Target Genes and Luciferase Reporter Assay

The potential target genes downstream of the candidate lncRNA were predicted using Starbase 2.0 software (http://starbase.sysu.edu.cn/starbase2/index.php) and the TargetScan (www.targetscan.org/vert_71/) database.

The full fragments of the candidate lncRNA or its mutant containing the putative miRNA-binding sites were synthesized and cloned downstream of the firefly luciferase gene in pGL3 plasmids (Promega Corporation, Madison, WI, USA), and were termed the pGL3-candidate lncRNA-wild type (Wt) and pGL3-candidate lncRNA-mutant (Mut). Eca109 and Eca109R cells were maintained in 96-well plates and co-transfected with 400 ng of the constructed luciferase reporter plasmids, 50 ng of *Renilla* luciferase reporter vector and 50 nM of the putative miRNA mimic, miR-con, or putative miRNA-vector using Lipofectamine 3000^TM^ (Thermo Fisher Scientific, Inc.). Cells were harvested at 48 h after transfection, and luciferase activity was determined using a Dual Luciferase Reporter Assay Kit (Promega Corporation). *Renilla* luciferase activities were used as the internal control for the normalization of firefly luciferase activity.

### *In vivo* Experiments

The animal experiments were approved by the Animal Care and Use Committee of Fujian Medical University Union Hospital and were performed in accordance with the Institutional Guide for the Care And Use Of Laboratory Animals. Lentiviral vector [Lenti-short hairpin (sh)-candidate lncRNA] for stable silenced expression of the candidate lncRNA was obtained from Shanghai GenePharma Co., Ltd. (Shanghai, China) and transfected into Eca109/Eca109R cells. The success of transfection was detected by PCR and the survival of the cells was determined by an MTT assay. Then, equal numbers of siRNA-candidate lncRNA-transfected Eca109, NC and control cells were implanted into 8-week old nude mice (*n* = 5 per group; Model Animal Research Center of Nanjing University) by subcutaneous injection.

At two weeks after the injection (to allow for tumor growth), the tumors were irradiated by X-ray at 10 Gy. Tumor size was measured every 3 days with a caliper, and tumor volume was calculated according to the following formula: Volume = (length x width^2^)/2. All mice were sacrificed on day 42 after inoculation. The resected tumor masses were harvested for subsequent weight measurements. A growth curve was constructed to determine tumor radiosensitivity and the effect of the siRNA of the candidate lncRNA on tumorigenicity in nude mice was analyzed.

### Statistical Analysis

The overall survival data was analyzed using SPSS software 23.0 (IBM Corp., Armonk, NY, USA). Survival curves were established through the Kaplan-Meier method and compared by a log rank test.

A multivariable analysis of patient demographic and clinical parameters (gender, age, ECOG score, tumor location, clinical T and N stages, the radiotherapy doses for GTV and CTV, and the tumor response to treatment) was performed using the Cox proportional hazards model.

Experimental data are presented as $\bar{\text{x}}\pm \text{s}$ from independent experiments performed in triplicate. For comparisons, paired or independent Student's *t*-tests, Chi-square tests or ANOVA with *post hoc* tests (Tukey's) were performed. ROC curves were used for selecting an optimal cut-off point for each test and for comparing the accuracy of diagnostic tests. Two-tailed *P* < 0.05 (^*^P < 0.05, ^**^P < 0.01, ^***^P < 0.001) was considered to indicate a statistically significant difference.

## Results

### Patient Characteristics, Treatment Response, and Survival

Between July 2015 and March 2017, a total of 41 ESCC patients treated with RT combined with chemotherapy were enrolled in the present study. After RT, a total of 4 patients achieved CR, 19 patients reached PR, 9 patients maintained SD and 9 cases had PD. There were no significant differences between radiosensitive (4 CR and 19 PR) and radioresistant (9 SD and 9 PD) patients regarding the distributions of gender, age, ECOG score, tumor location, and clinical stage (Table 2).

**Table 2:** Clinicopathological characteristics of the entire cohort of 41 patients with ESCC.

**Characters**	**Radiosensitive**	**Radioresistant**	**Total**	***p***
Gender				0.706
Male	17	15	32
Female	6	3	9
Age (range)	61 (47-70)	63 (47-70)	61 (47-70)	0.406
ECOG score				0.767
0	13	11	24
1	10	7	17
Tumor location				0.515
Cervical	4	3	7
Upper	5	6	11
Middle	11	8	19
Lower	3	1	4
T stage				0.112
2	1	1	2
3	11	3	14
4	11	14	25
N stage				0.164
0	2	0	2
1	14	8	22
2	7	10	17
M stage ^a^				0.542
0	20	15	35
1	3	3	6
Clinical stage ^b^				0.112
II	1	1	2
III	11	3	14
IV	11	14	25
GTV (cGy, range)	6000 (4000–6600)	6000 (5040–6600)	6000 (4000–6600)	0.128
CTV (cGy, range)	5040 (4000–5040)	5040 (4500–5040)	5040 (4000–5040)	0.300
IC				0.574
None	7	8	15
PF	5	2	7
TL	1	0	1
TP	10	8	18

*There were no significant differences between radiosensitive and radioresistant patients regarding the distributions of gender, age, ECOG score, tumor location and clinical stage. ECOG, eastern cooperative oncology group; GTV, gross tumor volume; CTV, clinical target volume; PF, platinum plus fluorouracil; TP, platinum plus taxane; a, M1 means Supraclavicular lymphatic node metastasis; IC, Induction chemotherapy; b, according to the 7th AJCC TNM staging system*.

### Differential Expression of lncRNAs Potentially Correlated With Radiosensitivity

**Figure 1:**
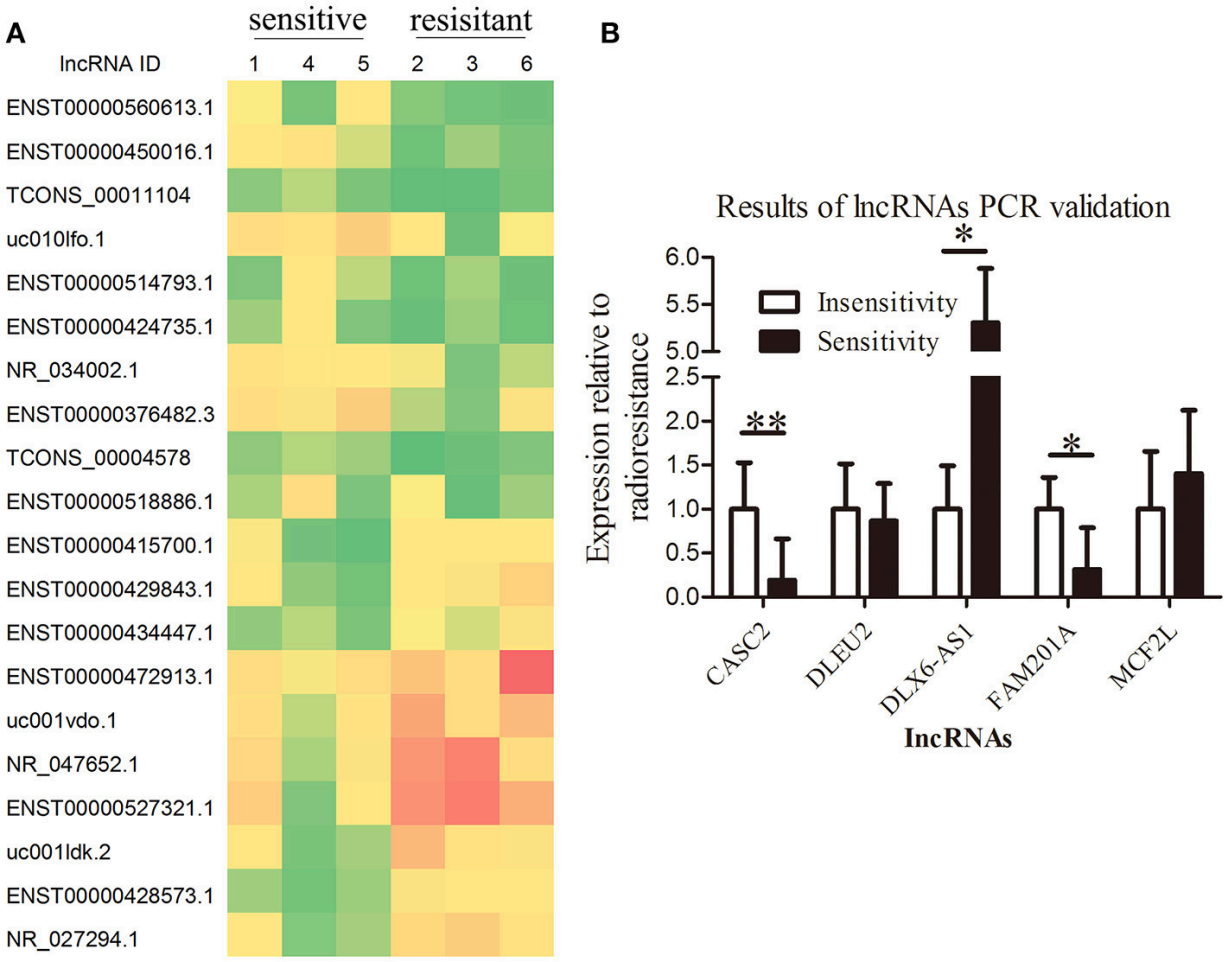
Overexpression of lncRNA FAM201A is highly correlated with the radiosensitivity of ESCC and is associated with poor survival. **(A)** A heatmap presenting the gene expression levels in RNA samples isolated from three radiosensitive and three radioresistant ESCC tumor tissues by microarray assays. **(B)** Differential expression of the potential lncRNAs related to radiosensitivity (CASC2, FAM201A, DLEU2, DLX6-AS1, and MCF2L-AS1) in radiosensitive (*n* = 20) and radioresistant (*n* = 15) ESCC tumor tissues by reverse transcription-quantitative polymerase chain reaction. *P < 0.05, **P < 0.01.

A total of 113 aberrantly expressed lncRNAs were identified in the microarray analysis using three radiosensitive ESCC tumor tissues and three radioresistant ESCC tumor tissues, of which 71 lncRNA transcripts were upregulated (fold change >2, *P* < 0.05) and 42 lncRNA transcripts were downregulated (fold change < 0.5, *P* < 0.05) in the radiosensitive ESCC tumor tissues when compared with the radioresistant ESCC tumor tissues. The lncRNAs CASC2, FAM201A, DLEU2, DLX6-AS1, and MCF2L-AS1 were considered to be the potential lncRNAs related to radiosensitivity when analyzed using GeneSpring Software 12.6 (Agilent Technologies, Inc.) (Figure 1A, Supplementary File 1).

Tumor tissues from the remaining 35 enrolled patients (20 radiosensitive patients and 15 radioresistant patients, respectively) were collected to detect the expression of the lncRNAs CASC2, FAM201A, DLEU2, DLX6-AS1, and MCF2L-AS1 by RT-qPCR. The results revealed that the differential expression of CASC2, FAM201A, and DLX6-AS1 between the radioresistant and radiosensitive groups were significantly different, while the difference in the DLEU2 and MCF2L-AS1 expressions were not significantly different when comparing the groups (Figure 1B; Supplementary File 2).

### FAM201A Is a Novel lncRNA With a Potential Function in the Radiosensitivity and Survival of ESCC

**Figure 2:**
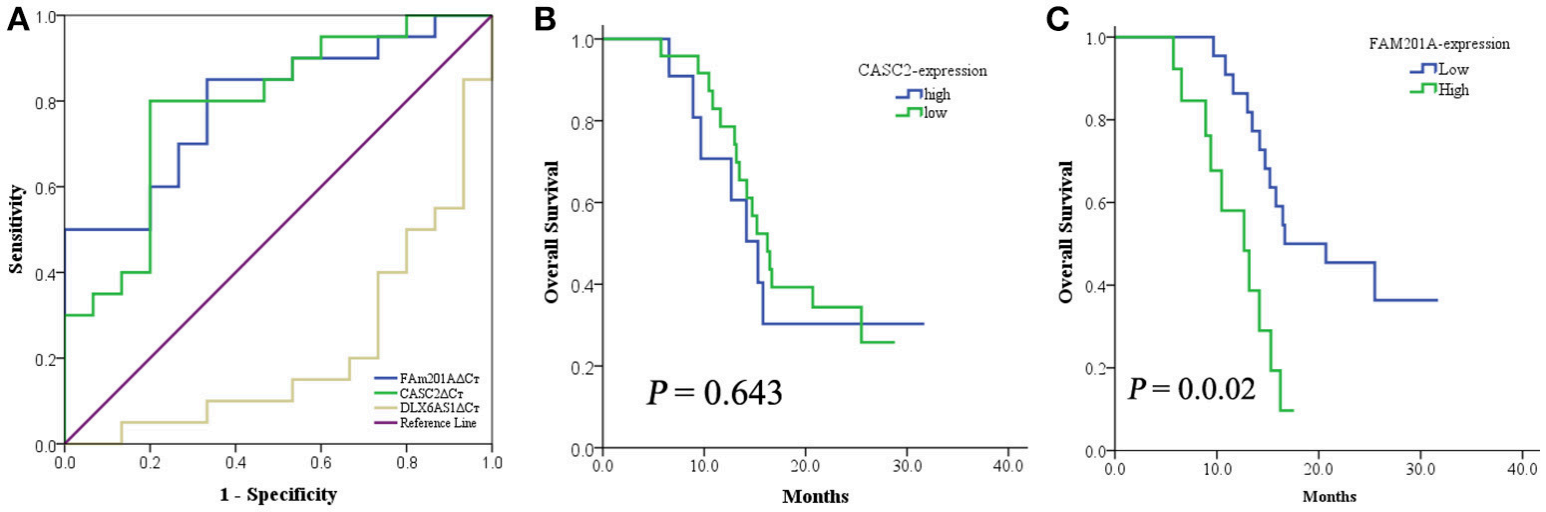
**(A)** The ROC curve of lncRNA CASC2, FAM201A, and DLX6-AS1. When compared with the lncRNA DLX6-AS1, FAM201A, and CASC2 yielded a superior AUC with specificity and sensitivity for distinguishing radiosensitive ESCC tumor tissues from radioresistant ESCC tumor tissues. **(B)** The 1-year OS rate between patients with low- (*n* = 24) and high-expression (*n* = 11) of CASC2, was not different. **(C)** The 1-year OS rate between patients with low- (*n* = 22) and high-expression (*n* = 13) of FAM201A was significantly different (*P* = 0.001).

Based on above data, the ROC curve of the lncRNAs CASC2, FAM201A, and DLX6-AS1 was applied to identify the lncRNA that was the most correlated to radiosensitivity and survival using the area under curve (AUC) were 0.783 (95%CI: 0.609–957, *P* = 0.005), 0.817 (95%CI: 0.673–960, *P* = 0.002), and 0.340 (95%CI: 0.150–530, *P* = 0.110); respectively. Compared with the lncRNA DLX6-AS1, FAM201A, and CASC2 yielded a superior AUC with specificity and sensitivity for distinguishing radiosensitive ESCC tumor tissues from radioresistant ESCC tumor tissues (Figure 2A).

CASC2 was associated with short-term response to RT but not with survival, while FAM201A was correlated with both the short-term response and survival (Figures 2B,C). This indicated that FAM201A, as opposed to CASC2, may be a suitable biomarker of ESCC treated with RT.

To analyze whether FAM201A functions as a biomarker for radiosensitivity and survival in ESCC or not, the maximum Youden index method (Fluss et al., 2005) was performed to establish the cutoff value of FAM201A in the ROC curve. A total of 22 patients were termed as FAM201A-low with an average ΔCt expression value of 6.155, whereas, the remaining 13 patients, named the FAM201A-high expression group, had an average ΔCt expression value of 8.437 (Supplementary File 3).

Compared with the FAM201A-low group, the FAM201A-high group exhibited a poorer short-term response to RT and lower survival time. However, neither high or low FAM201A expression was correlated with tumor stage, regardless of whether it was T or N stage (Table 3). Furthermore, univariate and multivariate analysis indicated that FAM201A was the only independent risk factor for survival (OR, 0.642; 95% CI, 0.4668–0.885; *P* = 0.007). These data suggested that FAM201A could be a robust molecular marker for predicting RT sensitivity and survival in patients with ESCC.

**Table 3:** Treatment results in the high and low FAM201A expression groups.

**Variable**	**Low FAM201A expression, *n***	**High FAM201A expression, *n***	**Total *n***	***P*-value**
T stage				0.161
2	0	2	2
3	11	5	16
4	11	6	17
N stage				0.998
0	3	2	5
1	10	6	16
2	7	4	11
3	2	1	3
M stage				0.388
0	20	13	33
1	2	0	2
Tumor response, *n* (%)				0.001
CR	1	0	1
PR	17	2	19
SD	3	6	9
PD	1	5	6
Pattern of failure, *n*				0.177
Locoregional alone	9	4	13
Locoregional and distant	0	2	2
Distant alone	4	4	8
1-year overall survival rate (%)	45.5	9.7		0.002

*The level expression of FAM201A was not correlated with the tumor stage, whatever in term of T stage or N stage. Compared with low expression FAM201A, patients with high expression of FAM201A resulted in poorer short-term response to RT. CR, complete response; PR, partial response; SD, stable disease; PD, progressive disease*.

### FAM201A Regulated Radiosensitivity *in vitro*

**Figure 3:**
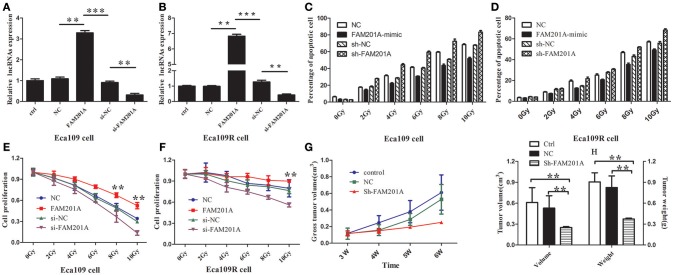
Reverse transcription-quantitative polymerase chain analysis confirmed the efficiency of transfected **(A)** Eca109 or **(B)** Eca109R cells with si-FAM201A and FAM201A-mimic. In both **(C)** Eca109 or **(D)** Eca109R cancer cells transfected with si-FAM201A or FAM201A-mimic, the percentage of apoptotic cells in each line increased with the increasing X-ray irradiation dose. In contrast to the levels of apoptosis, cell proliferation decreased with increasing radiation doses in **(E)** Eca109 or **(F)** Eca109R. The effect of shFAM201A on Xenograft tumor survival was also evaluated: **(G)** tumor survival curve, **(H)** tumor volume and weight. **P < 0.01, ***P < 0.001.

Based on the above results, the effects of FAM201A regulated radiosensitivity in ESCC cancer cells were further explored by performing an X-ray irradiation experiment using Eca109/Eca109R cells transfected with si-FAM201A and FAM201A-mimic (Figures 3A,B; Supplementary File 4).

The results revealed that the survival rates of both Eca109 and Eca109R cells decreased with the increasing X-ray irradiation dose, and the percentage of apoptotic cells in each line increased with the increasing X-ray irradiation dose (Figures 3C,D; Supplementary File 4). The decrease in survival was more pronounced with the increase in X-ray irradiation dose in ECA109 cells when compared with ECA109R cells, demonstrating that the Eca109R cells were more resistant to X-ray irradiation.

In Eca109 cells, when compared with the control cells, FAM201A-mimic exhibited a significant promotion in cell proliferation, while si-FAM201A exhibited a significant increase in proliferation inhibition, indicating that for Eca109 cells, upregulated FAM201A expression likely resulted in cell radioresistance to X-rays (Figure 3E; Supplementary File 4).

In Eca109R cells, when compared with the control cells, si-FAM201A exhibited a significant inhibition of cell proliferation, while FAM201A-mimic did not exhibit the increased cell proliferation that was observed in ECA109 cells, indicating that the expression level of FAM201A in Eca109R cells was already at a high level, and thus, further elevation of FAM201A expression was not possible to enhance its radioresistance. These results indicated that, whether in cases of intrinsic or acquired radioresistance, si-FAM201A may enhance ESCC cell radiosensitivity, which may therefore be a novel effective target strategy for sensitizing ESCC to radiotherapy (Figure 3F; Supplementary File 4).

### FAM201A Knockdown Enhanced the Radiosensitivity of ESCC *in vivo*

To confirm the efficacy of si-FAM201A on radiosensitivity *in vivo*, a xenograft tumor mouse model was established. A total of 15 mice with similar weights and dates of birth were selected in the present study (male: female = 8:7). When compared with the control groups, FAM201A knockdown (sh-FAM201A) significantly blocked tumor growth (decreased tumor volume and weight), suggesting that the silenced FAM201A expression enhanced radiosensitivity, thereby confirming that FAM201A could induce radiosensitivity *in vivo* (Figures 3G,H; Supplementary File 4).

### FAM201A Negatively Regulated the Expression of miR-101

Using Starbase 2.0, miR-101 and miR-590 were predicted to have complementary base pairings with FAM201A. Accordingly, luciferase reporter vectors containing the Wt or a Mut FAM201A binding site were established and co-transfected with miR-101 into Eca109 cells. The same process was performed for miR-590.

**Figure 4:**
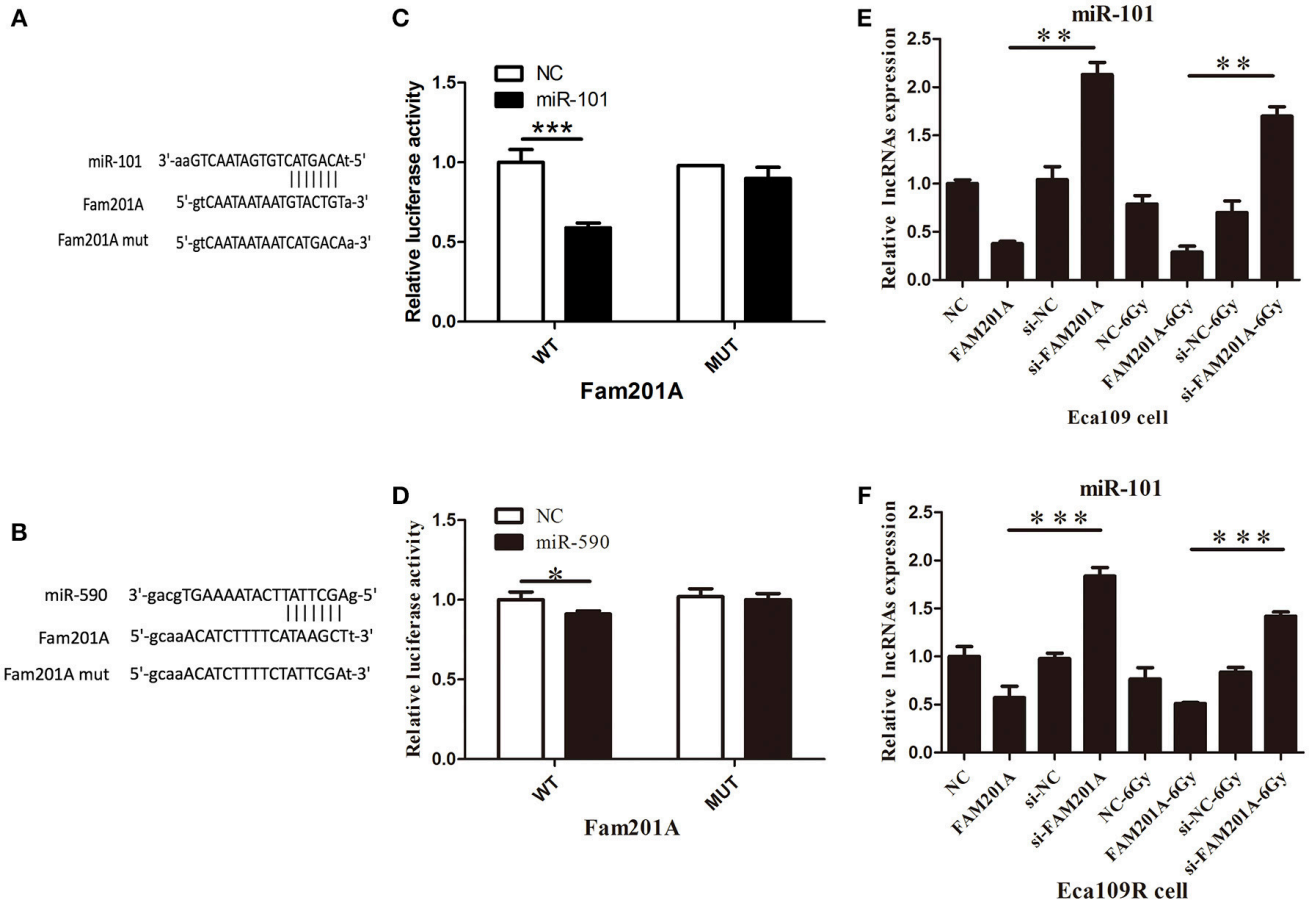
**(A)** miR-101 and **(B)** miR-509 were predicted to have complementary base pairings with FAM201A. The relative luciferase activity of the wild-type and mutated FAM201A were compared between **(C)** miR-101 and **(D)** miR-590. miR-101 expression was negatively regulated by FAM201A in **(E)** Eca109 and Eca1 **(F)** 109R cells. *P < 0.05, **P < 0.01, ***P < 0.001.

The results demonstrated that the ectopic expression of miR-101 was markedly suppressed by co-transfection with the FAM201A mutant sequence in the Eca109 cell luciferase activity reporter assay. However, neither pGL3-FAM201A-Wt reporter nor pGL3-FAM201A-Mut transfection in Eca109 cells affected miR-590 expression (Figures 4A,B; Supplementary File 5).

### FAM201A Upregulated ATM and mTOR Expression by Acting as a miR-101 Sponge

To further evaluate the regulatory relationship between FAM201A and miR-101, Eca109 cells were transfected with si-FAM201A and FAM201A-mimic sequences and matched controls. The results revealed that miR-101 expression was significantly downregulated in FAM201A-mimic Eca109/Eca109R cells, and was notably upregulated in si-FAM201A-transfected Eca109/Eca109R cells (Figures 4C,D). Taken together, these results indicated that FAM201A suppressed the expression of miR-101 (Supplementary File 6).

**Figure 5:**
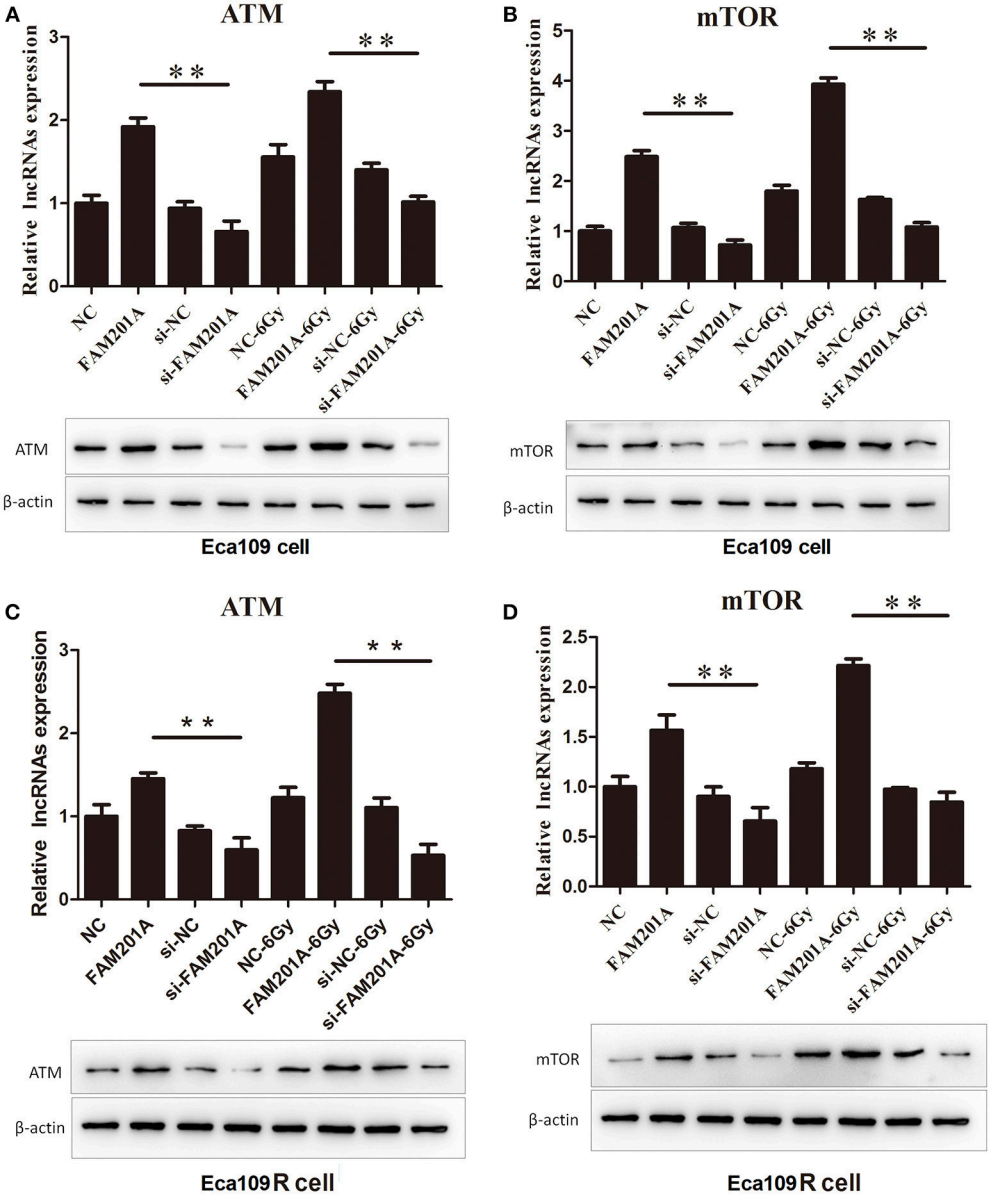
Effects of overexpressed- and si-FAM201A on the expression of miR-101, ATM and mTOR in **(A, B)** Eca109 and **(C, D)** Eca109R cells before and after X-ray irradiation. Western blotting validation of ATM and mTOR in Eca109 and Eca109R cells. **P < 0.01.

Using TargetScan, ATM and mTOR were predicted to be the downstream targets of miR-101. In Eca109/Eca109R cells, the expression of ATM and mTOR was increased while that of miR-101 was decreased in FAM201A-mimic cells when compared with control cells. When FAM201A expression was decreased, the expression of ATM and mTOR was downregulated while that of miR-101 was increased. Compared with non-irradiated cells, the expression of ATM and mTOR increased after X-ray irradiation. Western blotting confirmed the results of PCR (Figure 5; Supplementary File 6).

## Discussion

The earliest study on lncRNAs associated with radiosensitivity in ESCC was reported by Tong et al. in 2014 (Tong et al., 2014). In this study, they revealed that, when compared with normal para-carcinoma tissue, tumor tissues with a low expression of lncRNA LOC285194 exhibited a larger tumor size, poorer histological grade, had an advanced TNM stage, more lymph node and distant metastases, and was significantly negatively correlated with the pathological response to RT than the LOC285194-high group. Subsequently, researchers have revealed another three lncRNAs related to ESCC radiosensitivity, including BOKAS (Zhang et al., 2015), MALAT1 (Li et al., 2016), and AFAP1-AS1 (Zhou et al., 2016). However, clinical trials for evaluating such lncRNAs related to ESCC radiosensitivity are lacking as the mechanism for how lncRNAs regulate radiosensitivity has yet to be fully elucidated, and so no promising lncRNAs have been applied in the clinic.

In the present study, we identified that the lncRNA FAM201A contributed the most to the radioresistance of ESCC regardless of the tumor stage. The FAM201A gene is a 2.9 Kbp long gene located in genomic 9p13.1 (Humphray et al., 2004) that results in RNA transcripts without ORFs, which means that it has no protein-coding potential. FAM201A in human diseases has been reported crudely in Obsessive-compulsive disorder and Tourette's syndrome by Yu et al. (2015), while it was first mentioned in cancer (colorectal) by Matsumura et al. (2017). Recently, Huang et al. revealed that the biofunction of FAM201A was involved in the development of Osteonecrosis of the femoral head (Huang et al., 2018). However, the molecular mechanism of lncRNA FAM201A function has not been studied. To the best of our knowledge, the present study was the first to report on the correlation of FAM201A with ESCC radiosensitivity and to investigate its potential molecular mechanism, in order to elucidate whether it may be a biomarker for the prognosis and prediction of the patient's response to RT.

The results revealed that patients with FAM201A overexpression had poorer radiosensitivity and inferior survival. Conversely, lower FAM201A expression in ESCC was associated with improved radiosensitivity and a good prognosis, indicating that lnc-FAM201A may serve as a predictor of radiosensitivity in ESCC.

Subsequently, we performed experiments *in vitro* and *in vivo* to confirm the functions of FAM201A. *In vitro*, the overexpression of FAM201A was demonstrated to promote Eca109 cell proliferation; while decreasing FAM201A expression inhibited cell proliferation. The difference in radioresistance following the overexpression of FAM201A in Eca109 and Eca109R cells indicated that FAM201A upregulation likely resulted in cell radioresistance to X-rays irradiation. In addition, the similar levels of radiosensitivity following the reduction in FAM201A expression in Eca109 and Eca109R cells suggested that si-FAM201A may enhance the radiosensitivity of both intrinsically and acquired-radioresistant tumor cells, indicating that siFAM201A may serve as an effective sensitizing molecular strategy for ESCC. *In vivo*, when compared with control groups, FAM201A knockdown significantly blocked xenograft tumor growth (decreased tumor volume and weight), which confirmed that siFAM201A was able enhance radiosensitivity.

Recently, a competing endogenous RNAs hypothesis proposed that lncRNAs may exert their biological function by acting as a molecular sponge for miRNAs, in turn leading to derepression of miRNA targets (Tay et al., 2014). To explore the molecular mechanism of FAM201A-modulated radiosensitivity in ESCC, we used the online software Starbase 2.0 to predict the downstream target genes, and found that miR-101 and miR-590 had complementary base pairings with FAM201A. Only miR-101, and not miR-590, was observed to directly interact with FAM201A, as determined by the luciferase reporter assay. The qPCR analysis further demonstrated that FAM201A overexpression downregulated miR-101 expression while si-FAM201A transfection upregulated miR-101. These results suggested that FAM201A may modulate target gene expression by serving as a “sponge” for miR-101 (Kung et al., 2013).

Further, the role of miRNAs usually depends on what genes they target. The TargetScan analysis showed that ATM and mTOR were the targets of miR-101. Furthermore, qPCR revealed that overexpression of FAM201A leads to the downregulation of miR-101, the upregulation of ATM and mTOR, and resulted in radioresistance; however, depletion of FAM201A led to the upregulation of miR-101, downregulation of ATM, and mTOR, and resulted in radiosensitivity. Additionally, western blotting confirmed these PCR results.

ATM is the major repair protein involved in the homologous recombination repair (HRR) of ionizing radiation-induced double-strand breaks (RI-DSB). ATM deficiency leads to HRR disorders, increased apoptosis and radiosensitivity (Cliby et al., 1998; Cuddihy and Bristow, 2004; Hammond and Muschel, 2014). Therefore, we hypothesize that FAM201A may regulate ESCC radiosensitivity via a “FAM201A-miRNA101-ATM-HRR” axis.

HRR occurs only in the S and G2 phases of DNA replication, due to the requirement of homologous sister chromatids as a template (Pâques and Haber, 1999). DSBs during the absence of homologous sequence chromosomes requires non-homologous end joining (NHEJ) to achieve DNA repair, which is a repair function performed throughout the cell cycle and was initially considered to be the primary mechanism of RI-DSB repair (Branzei and Foiani, 2008; Beucher et al., 2009). Yan et al. (2010) reported that miR-101 regulates the radiosensitivity of cells by regulating the DNA-dependent protein kinase catalytic subunit, an important member of the NHEJ machinery, via mTOR. Therefore, we hypothesize that lncRNA-FAM201A may also modulate cell ionizing radiosensitivity via a “FAM201A-miR-101-mTOR-NHEJ” axis. In future research, we will focus on the upstream mechanism underlying FAM201A upregulation in regulating ESCC radiosensitivity.

## Conclusions

In conclusion, the present study revealed that lncRNA FAM201A may be a potential biomarker for predicting radiosensitivity and prognosis, as well as a therapeutic target for enhancing cancer radiosensitivity in ESCC. FAM201A contributed to radioresistance through a FAM201A-miR-101-ATM/mTOR regulatory network in ESCC. However, the upstream mechanism for FAM201A upregulation in regulating ESCC radiosensitivity requires further study.

## Ethics Statement

This study was subject to approval by the Fujian Medical University Union Hospital Institutional Review Board (No. 2014KY001). All patients signed an informed consent prior to treatment, and all information was anonymized and deidentified prior to its analysis.

## Authors Contributions

MC, PL, YT, JC, and JL conceived the study, manuscript, and statics analysis. YL, XiaL, MS, XiqL, and AL assistance with collecting clinical data. RY, WN, XZ, YC, and LZ provided assistance with study design and revisions of the manuscript. All authors read and approved the final manuscript.

### Conflict of Interest Statement

The authors declare that the research was conducted in the absence of any commercial or financial relationships that could be construed as a potential conflict of interest.

## Supplementary Material

File S1 Results of lncRNA chip microarray.

File S2 Results of PCR of candidate lncRNAs.

File S3 FAM201A as a novel lncRNA with a potential function in radiosensitivity.

File S4 Validation of FAM201A *in vitro* and *in vivo*.

File S5 The results of the luciferase assay.

File S6 Overexpression of FAM201A and its effect on miR-101/ATM/mTOR in Eca109 and Eca109R cells.

## Abbreviations

ATM: ataxia telangiectasia mutated
AUC: area under the curve
CASC2: cancer susceptibility candidate 2
CR: complete response
CTV: clinical target volume
DLEU2: deleted in lymphocytic leukemia 2
DLX6-AS1: DLX6 antisense RNA 1
DNA: deoxyribonucleic acid
DNA-PKcs: DNA-dependent protein kinase catalytic subunit
ECOG: Eastern Cooperative Oncology Group
ESCC: esophageal squamous cell carcinoma
FAM201A: family with sequence similarity 201-member A
GTV: gross tumor volume
HRR: homologous recombination repair
IC: Induction chemotherapy
lncRNA: long non-coding RNA
MCF2L-AS1, MCF2L antisense RNA 1. mTOR: mammalian target of rapamycin
MTT, 3-(4, 5)-dimethylthiahiazo(-z-y1)-3: 5-di-phenytetrazoliumromide
miR: microRNA
NHEJ: non-homologous end joining
OS: overall survival
PCR: polymerase chain reaction
PD: progression of disease
PR: partial response
RT-qPCR: reverse transcription-quantitative polymerase chain reaction
RI-DSB: radiation-induced double-strand breaks
RNA: ribonucleic acid
ROC: receiver operating characteristic
RT: radiotherapy
SD: stable disease
sh-RNA: short hairpin RNA
siRNA: small interfering RNA
TP: platinum compound plus taxane.
