# Supplementary figures and images for: Long Noncoding RNA FAM201A Mediates the Radiosensitivity of Esophageal Squamous Cell Cancer by Regulating ATM and mTOR Expression via miR-101
Source: Front Genet. 2018 Dec 5;9:611. doi: 10.3389/fgene.2018.00611 (PMC6292217; doi:10.3389/fgene.2018.00611)

## Slide 1
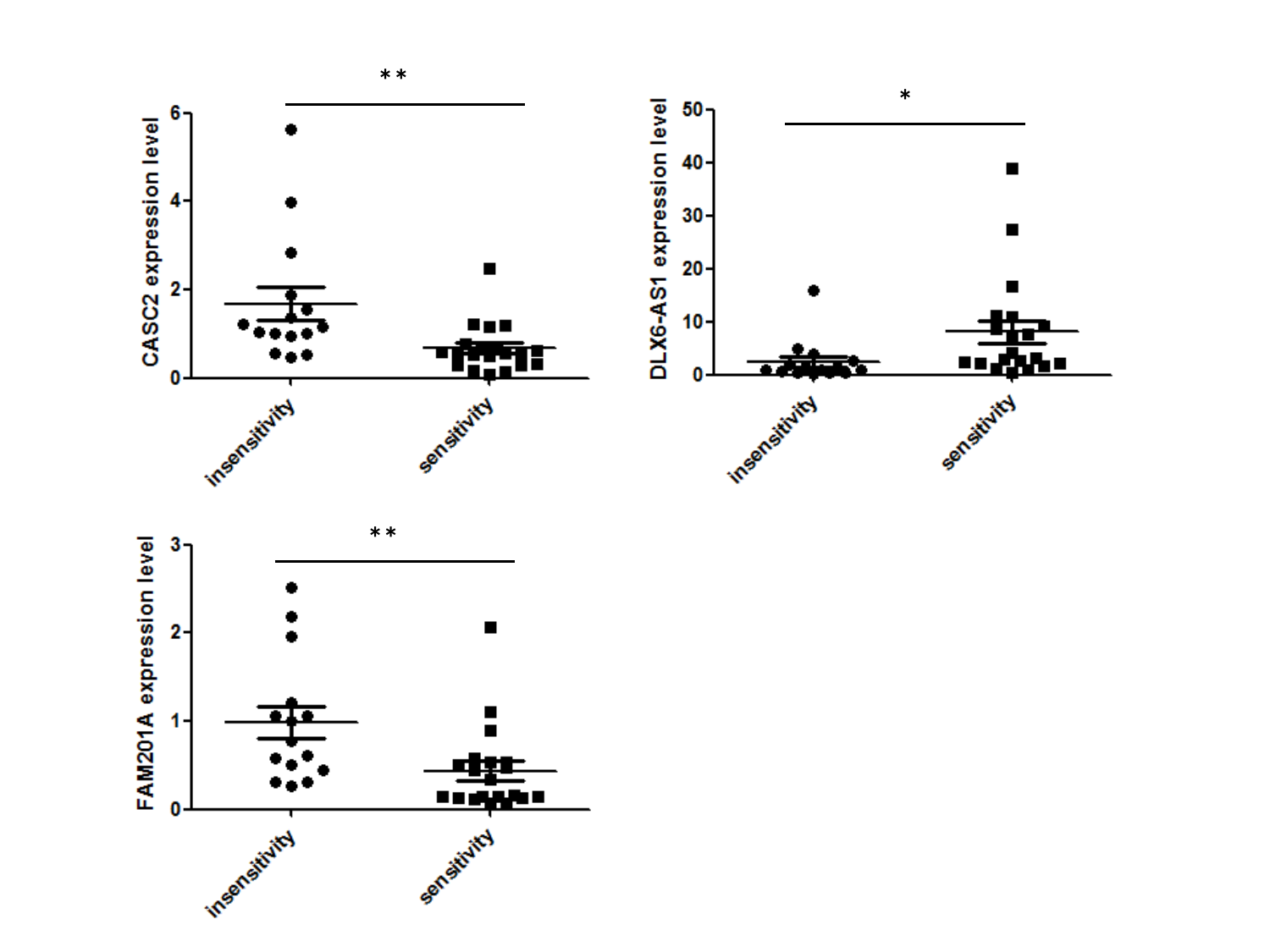

**
*
**

Supplement: File S1 — Results of lncRNA chip microarray. [file Data_Sheet_1.ZIP › Supplementary File/Supplementary file 2--Results of PCR of candidate lncRNAs/PCR of three potential lncRNAs for further study.pptx]

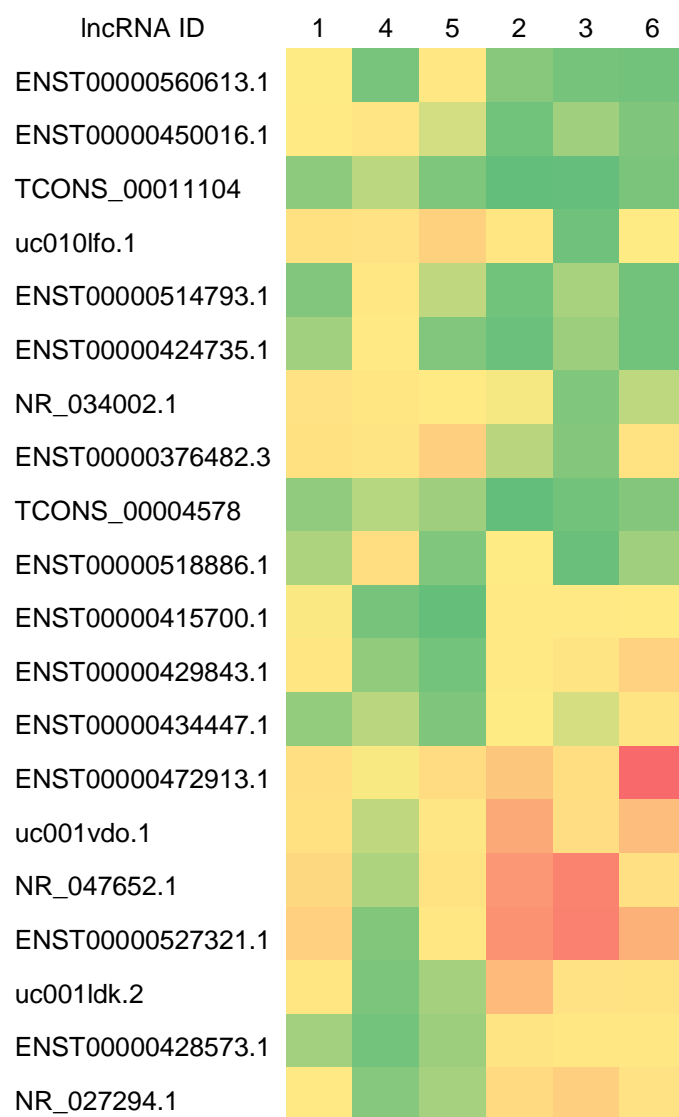

Supplement: File S1 — Results of lncRNA chip microarray. [file Data_Sheet_1.ZIP › Supplementary File/Supplementary file 1--Results of lncRNA chip microarray/Heatmap.pdf]
