# Supplementary material for: Long Noncoding RNA FAM201A Mediates the Radiosensitivity of Esophageal Squamous Cell Cancer by Regulating ATM and mTOR Expression via miR-101
Source: Front Genet. 2018 Dec 5;9:611. doi: 10.3389/fgene.2018.00611 (PMC6292217; doi:10.3389/fgene.2018.00611)
Supplement: File S1 — Results of lncRNA chip microarray. [file Data_Sheet_1.ZIP › Supplementary File/Supplementary file 2--Results of PCR of candidate lncRNAs/Reports of qRT-PCR.doc]

**定量PCR检测实验报告**

**1 主要试剂与仪器**

SYBR^®^ Premix Ex Taq（Takara，RR820A，即SYBR green溶液）

PrimeScript^™^ RT reagent Kit with gDNA Eraser（Takara，RR047A，即逆转录试剂盒）

RNAiso Plus（Takara，9108，即Trizol）

氯仿（Trichloromethane，上海阿拉丁生化，C128130）

异丙醇（isopropanol，上海阿拉丁生化，I112011）

乙醇（ethanol，上海阿拉丁生化，E111989）

DEPC（Diethyl pyrocarbonate，上海阿拉丁生化，D105557）

Stepone plus 荧光定量PCR（ABI）

PCR仪（ABI，[Veriti](http://www.baidu.com/baidu.php?url=0f0000aPftK-mFJ6dIPxuoxLoQiwzrT0QLzsZxlqC-xMX4upZWlzS4xS1l7XW1OiItPbviUdRq8ry2vMNx0_onQw0QV11VaQNZtYbJEIVgAEDCpryzvIH8izBh_LRZF4IRWOBZ4674RgBS6PswpVJpOfSy7_BMOQmFDvgr_EJ2UDXbFAk6.7Y_imLqap5bMlD1FWCRDsdXAjWYe_bD5_eGSW-WknUPMN5YTVZCmlIvHF3qhl1ugblGSW-9hlA2OZFktUQrMx_LIROwz3lQZgKPwmJCRnXZWemcrj63529RhUieySrGnzccmCrAJaGv-n77SVrXr7lmCRsm3uC27iuVQPZ5jC9CGhW11FuCYZlR4LkwYF_A-nNara9RhUi1w1slcp2uC35CHnS6O5fJHPGuC43IDP7nL5jkSEsOx4mX5z9B1L3_tpIrXSeV1lbkF1_SELSyxZjMYqVj5u342lOZr1xjIM_zxj3eo5UleS1It5o3SvEjblmSgV3cTyZWtEUSEjbo3qreF9zNePhZFgg34r5WuuYeqhejbos42SMIbzX5u8_vN47BmLIPMHuuvUrg_4TXGmuCyr1xggyC.U1Yk0ZDqmyFGFHcsRDPi0ZKGm1Yk0ZfqmyFGFHcsIhNzpgwGFHcsTAPzY_T0pyYqnWcd0ATqTZnz0ZNG5yF9pywdUAY0TA-b5HD40APGujYdPfKVIjYknjDLg1DsnH-xnH0YP7t1PW0k0AVG5H00TMfqPHTz0ANGujYkPjRYg1D4njnvg1cknjbzg1cdnHTL0AFG5HcsP7tkPHR0UynqP101PjDkPWfYg1Tsn1fknHmYPdtznjDdPjRLrjndg100TgKGujYs0Z7Wpyfqn0KzuLw9u1Ys0A7B5HKxn0K-ThTqn0KsTjYdP16LnH0kP1m0UMus5H08nj0snj0snj00Ugws5H00uAwETjYs0ZFJ5H00uANv5gKW0AuY5H00TA6qn0KET1Ys0AFL5Hm0UMfqn0K1XWY0IZN15HD1rHb4PWc4PWDdPjRzn1f3nWDY0ZF-TgfqnHRkn1R4P101PW0LPsK1pyfquy7-nHcdmHDsnj0snWbvufKWTvYqfbfdPRnzn16kPbuKwHmsffK9m1Yk0ZK85H00TydY5H00Tyd15H00XMfqn0KVmdqhThqV5HKxn7tknjfvg100uA78IyF-gLK_my4GuZnqn7tsg1Kxn0Ksmgwxuhk9u1Ys0AwWpyfqn0K-IA-b5iYk0A71TAPW5H00IgKGUhPW5H00Tydh5HDv0AuWIgfqn0KhXh6qn0Khmgfqn0KlTAkdT1Ys0A7buhk9u1Yk0Akhm1Ys0APzm1Y3PjDs&ck=3408.4.0.0.0.213.183.0&shh=www.baidu.com&sht=56060048_4_pg)）

Nano drop 2000（Thermo fisher）

**2 实验原理**

SYBR Green I是一种只与DNA双链结合的荧光染料。当它与DNA双链结合时，发出荧光；从DNA双链上释放出来时，荧光信号急剧减弱。因此，在一个体系内，其信号强度代表了双链DNA分子的数量。SYBR Green荧光染料法定量PCR的基本过程是：1、开始反应，当SYBR Green染料与DNA双链结合时发出荧光。2、DNA变性时，SYBR Green染料释放出来，荧光急剧减少。3、在聚合延伸过程中，引物退火并形成PCR产物。4、聚合完成后，SYBR Green染料与双链产物结合，定量PCR系统检测到荧光的净增量加大。

**3 实验方法**

2.1 将组织从-80℃冰箱中取出后，将组织放入无RNase的EP管中，加入1ml Trizol溶液，用研磨棒冰上研磨至无明显沉淀，冰上静置10min。

2.2 加入氯仿200ul，用力振摇15s，室温静置15min，离心（4℃,12,000g,15min）.

2.3 离心后液体分为三层，小心吸取上层无色液体移入一新的EP管中。

2.4 加入等体积异丙醇，上下颠倒混匀,室温静置5min,离心（4℃,12,000g,10min）。

2.5 去上清,沉淀加入75％[乙醇](http://shop.51atgc.com/index.php?app=search&cate_id=&keyword=???&Submit=????)1ml,轻微振荡15s，离心（4℃,7,500g,5min）。

2.6小心去上清，管内沉淀在超净台中鼓风静置干燥3-5min.最好是用枪头吸取上清,尽量除去。
 2.7加入50μl DEPC水溶解，nanodrop检测浓度，-80℃[冰箱](http://shop.51atgc.com/index.php?app=search&cate_id=&keyword=????&Submit=????)保存。

RNA纯度根据nano drop检测的浓度和OD230，OD260，OD280决定。

2.8逆转录

逆转录实验步骤按照逆转录试剂盒说明书操作（takara, RR047）具体逆转录体系如下：

去除基因组DNA。

| 试剂 | 体积 |
| --- | --- |
| 提取的总RNA | 6 µL |
| 基因组DNA污染清除试剂(gDNA Eraser) | 1 µL |
| 基因组DNA污染清除缓冲液(5×gDNA Eraser buffer) | 2 µL |
| DEPC水 | 1 µL |

注：按照以上方法在冰上进行反应液的配制，然后放置于室温，反应10min。

该部分就是根据上述体系将试剂混匀即可，由于是试剂盒，所以里面里面出现的试剂是试剂盒中的试剂。

逆转录PCR反应

| 试剂 | 体积 |
| --- | --- |
| 第一步反应液 | 10 µL |
| 反应缓冲液 5×Primescript Buffer | 4µL |
| 反转录酶 5×Primescript RT Enzyme Mix | 1µL |
| 反转录随机引物 RT Primer Mix | 4µL |
| DEPC水 | 1µL |

注：1、按照以上方法混合后，置于PCR仪中进行反转录，条件为37℃ 15 min，85℃ 5 s。

上述去除基因组和逆转录反应，是TAKARA PrimeScript^™^ RT reagent Kit with gDNA Eraser试剂盒中的试剂，根据试剂盒操作即可，就是将RNA同上述反应体系的试剂混匀后，放入PCR中根据所需的温度反应即可。

2.9荧光定量PCR

| 试剂 | 体积 |
| --- | --- |
| cDNA | 2µL |
| PCR上游引物（10µM） | 0.4 µL |
| PCR下游引物（10µM） | 0.4 µL |
| SYBR Green solution | 10 µL |
| 灭菌双蒸水 | 7.2 µL |
| 总量 | 20 µL |

反应条件：

| 95.0 ℃ | 10min |  |
| --- | --- | --- |
| 95.0 ℃ | 5 s | 40 cycles |
| 60.0 ℃ | 1min |  |
| 95.0 ℃ | 15 s | melt curve |
| 60.0 ℃ | 1 min |  |
| 95.0 ℃ | 15 s |  |

定量PCR根据上述反应体系加入各种反应试剂，SYBR green是混匀好的试剂，里面有Taq polymerase，dNTP，Sybr green染液，反应缓冲液。整个试剂配制体系在冰上配制，根据上述反应体系轻轻混匀后，将8连管用盖子盖好后，在离心机中瞬时离心将贴在管壁上的液体沉到底部。将8连管放入定量PCR检测，采用以上反应体系。PCR反应结束后，直接将8连管丢弃，直接根据仪器收集的数值对数据进行分析即可。

**4 实验结果**

图 放射敏感和不敏感的组织中CASC2，DLX6-AS1，FAM201A表达变化

从结果中可以看出，相对于射线不敏感的病例组织中，CASC2，FAM201A在射线敏感的病例组织中表达下调，而DLX6-AS1表达上调。
